# Smoking, Barriers to Quitting, and Smoking-Related Knowledge, Attitudes, and Patient Practices Among Male Physicians in China

**Published:** 2008-12-15

**Authors:** Marion Ceraso, Jane A. McElroy, Xiaodong Kuang, Peter M. Vila, Douglas E. Jorenby, Michael C. Fiore, Xueping Du, Ning Qian, Long Lu, Hongkun Ren

**Affiliations:** University of Wisconsin School of Medicine and Public Health; University of Missouri, Columbia, Missouri; University of Wisconsin School of Medicine and Public Health, Madison, Wisconsin; University of Wisconsin School of Medicine and Public Health, Madison, Wisconsin; University of Wisconsin School of Medicine and Public Health, Madison, Wisconsin; University of Wisconsin School of Medicine and Public Health, Madison, Wisconsin; Fuxing Hospital of the Capital University of Medical Sciences, Beijing, China; Fuxing Hospital of the Capital University of Medical Sciences, Beijing, China; Inner Mongolia Hospital, Hohhot, China; Inner Mongolia Hospital, Hohhot, China

## Abstract

**Introduction:**

Successful interventions to reduce the high rate of smoking among male physicians in China might contribute to reduction in tobacco use in the country overall. Better characterization of smoking, barriers to quitting, and smoking-related knowledge, attitudes, and patient practices in this physician population will help plan such interventions and provide baseline data to evaluate their effectiveness.

**Methods:**

A self-administered survey of smoking-related knowledge, attitudes, behaviors, and patient practices was conducted among health care professionals in 2 large teaching hospitals in China.

**Results:**

Of 103 male physicians, those who smoked (n = 51) had a more limited knowledge of smoking-related disease and were less likely to advise patients to quit smoking compared with nonsmoking physicians (n = 52). More than one-fourth (29%) of nonsmoking physicians accepted gift cigarettes, and these physicians were less likely to ask their patients about their smoking status than those who did not accept gift cigarettes. Seventy-five percent of smokers reported that their hospitals did not help them quit, and only 19% reported receiving training in how to help their patients quit.

**Conclusion:**

High rates of smoking, gifting of cigarettes, limited support for physician quitting, and limited training on cessation approaches may compromise the ability of male physicians in China to effectively treat their patients who smoke.

## Introduction

Smoking among physicians has been nearly eliminated in many countries (1). In China, however, smoking rates among male physicians remain high: an estimated 60% in the most recent population-based survey (2). This rate is consistent with rates of smoking among men in general in China: an estimated 61% in 1984 and 67% in 2002 (3,4). Tobacco use accounts for approximately 1 million premature deaths annually in China (5), and the proportion of deaths in men that can be attributed to tobacco use is expected to be 33% by 2030 if present smoking patterns continue (6).

Tobacco-control researchers have called for interventions to help physicians in China and other developing countries quit smoking and improve their treatment of patients who smoke (7-9). Designing and evaluating successful interventions require characterization of smoking patterns and personal, professional, and cultural factors related to smoking among Chinese physicians (10). Consequently, we designed this study to assess smoking status, barriers to quitting, and smoking-related knowledge, attitudes, and patient practices by using survey data collected from 103 male Chinese physicians in 2 hospitals.

## Methods

In 2006, we obtained permission from 2 large teaching hospitals — 1 in Hohhot, Inner Mongolia, and 1 in Beijing — to distribute 492 surveys to a convenience sample of 238 nurses and 254 physicians in selected departments. These departments — internal medicine, surgery, obstetrics/gynecology, pediatrics, neurology, orthopedics, family medicine, and radiology — were chosen because of our strong relationship with them. Participants were paid ¥8 (approximately US $1) for completing the survey. This study was approved by the Health Sciences Institutional Review Board of the University of Wisconsin School of Medicine and Public Health as well as the institutional review boards of the collaborating hospitals.

The anonymous, self-administered, 60-item questionnaire was developed based, in part, on the World Health Organization's Global Health Professionals Survey (11). The survey included information on demographics; smoking-related knowledge, attitudes, and behaviors; and patient counseling and treatment practices. Survey questions were translated into Chinese, and all questions were back-translated into English to verify accuracy of translation. Written responses were also back-translated into English for analysis.

Of the 492 surveys distributed, 416 (85%) were completed. Because almost none of the women we surveyed smoked, we limited our analysis to men. Of the 216 male physicians in the selected departments, 116 received a questionnaire and 103 (89%) completed it.

Smokers were defined as those who had smoked at least 100 cigarettes in their lifetimes and had smoked on at least 1 day in the month before the survey. Data were analyzed by using SPSS for Windows version 14.0 (SPSS Inc, Chicago, Illinois). The Fisher exact test was used to assess the relation between the dependent variable (smoking status) and independent variables (smoking-related knowledge, attitudes, and patient practices).

## Results

Mean age of our respondents was similar in both hospitals (37 years in Hohhot and 38 in Beijing), as were length of employment (11 years in Hohhot, 14 in Beijing) and percentage of smokers (44% in Hohhot, 55% in Beijing). Smoking was associated with less knowledge of smoking-related health risks, with the exception of the risk for lung cancer. The difference was particularly apparent in knowledge of the risks of exposing children to secondhand smoke (Table).

Fewer smokers than nonsmokers agreed that health care professionals should advise patients to quit or advised their smoking patients to quit. Less than one-fifth (19%) of male physicians reported receiving any training in smoking cessation approaches. Most smokers (75%) reported no offers of assistance from their hospitals to help them quit, although 57% reported having stopped smoking for at least a week.

Among nonsmokers, 29% always or sometimes accepted cigarettes when offered to them, and fewer of those who accepted cigarettes reported that they asked their patients about smoking status (71% vs 94%, *P* = .03). Among smokers, 84% accepted cigarettes as gifts. Forty-five percent of nonsmokers and 59% of smokers agreed that smoking was socially acceptable among medical professionals.

## Discussion

Our finding of high rates of smoking among male physicians in China, as has been reported previously (8,9,12,13), stands in stark contrast to the situation in many other countries where smoking has been virtually eliminated in the physician population (1). Physicians who smoked were significantly less likely than nonsmokers to agree that smokers are at increased risk for a number of diseases; they were also less likely to intervene with their smoking patients. These results confirm that smoking by physicians may compromise their ability to effectively treat patients who smoke (7,8,14).

Previous surveys of smoking among Chinese physicians have pointed to the need for efforts to decrease physician smoking (9,12) and increase intervention with patients who smoke (9,12,15). Of these studies, however, 1 is nearly a decade old (12), and another (15) was conducted among Hong Kong physicians only, a population that may have different social and cultural constraints than those experienced by mainland Chinese (16). Our survey builds on these previous studies by including additional data on the social context of smoking through questions on the gifting of cigarettes and the perceived acceptability of smoking among health professionals.

A better understanding of the social context for continued high rates of smoking among physicians in China is critical to developing interventions that reduce tobacco use in this population. We found that most smokers and almost half of nonsmokers agree that smoking is acceptable among medical professionals. This finding is less surprising in the context of previous reports that describe the gifting and sharing of cigarettes as a way to cement relationships between men in China (12,17-19). Chinese men, even medical professionals, experience strong pressures to smoke, and this pressure creates conflict in men who also feel pressure to quit (18,20). Refusing an offered cigarette has been described as an antisocial act in China (18,20).

The social pressure that reinforces and rewards male smoking is probably a barrier both to quitting and to advising patients to quit. Our finding that male physicians who did not smoke but who accepted cigarettes were less likely to ask their patients about smoking status may indicate that these nonsmokers are more susceptible to smoking-related social pressures that influence their personal and professional behavior. In other words, this susceptibility may be a reason that these nonsmoking physicians both accept cigarettes when offered and are less willing to address the issue of smoking with their patients.

Support and training for treating tobacco dependence has been described previously as critical for effective physician intervention with patients (15,20). Our study found, however, that the 2 hospitals in our survey, despite being teaching hospitals, did not substantially support physicians in their efforts to quit, nor did they train physicians in how to intervene with smoking patients. Other health professional groups in China may be facing similar issues. For example, in 1 large study, Chinese nurses cited lack of motivation among patients, heavy workload, lack of time, and lack of tobacco-cessation training as barriers to intervening with patients who smoked (21).

Our findings may be relevant for the development of strategies to reduce tobacco use among male physicians in China. First, approaches to counteract the role of cigarettes as gifts and relationship-builders should be considered (22,23). These might take the form of refusal-skills training or, as has been suggested elsewhere (22), the substitution of a nontobacco alternative. This recommendation may be relevant for strategies aimed at the general Chinese population as well. Second, the perceived acceptability of smoking among health professionals should be addressed and will probably require attention from the highest levels of medical leadership to change institutional and organizational professional codes to enforce smoke-free facilities and support quitting among providers. Third, training on how to implement clinical protocols for treating patients who smoke should be instituted for practicing physicians and as part of the medical school curriculum.

Some limitations should be considered in interpreting our results. Because all information in the study was self-reported, some bias in reporting may have occurred. We did not biochemically confirm smoking status. Our modest sample size constrained our analysis and limited the precision of our estimates. Although our findings were similar in 2 hospitals in distinct regions of China, the results may not be representative of the broader population of physicians in China.

Others have noted the inherent challenges in changing social norms that support smoking (18,24). Ideas suggested in this article that relate to the medical profession's role in tobacco control should be considered as a piece of a much larger effort. Multiple approaches will be required to denormalize tobacco use among men, including physicians, in China. Effective and culturally appropriate treatment strategies to help physicians quit and increased training to enable physicians to intervene effectively with their patients who smoke are urgently needed and should be a critical part of a comprehensive tobacco control plan for China.

## Figures and Tables

**Table.** Knowledge of Smoking Risks and Attitudes and Patient Treatment Practice Among Male Physicians by Smoking Status, China, 2006

**Knowledge, Attitude, or Practice Question**	No. of Smokers (%) (n = 51)^a^	No. of Nonsmokers (%) (n = 52)^a^	***P* Value**
**Lung cancer associated with smoking**			
Agree	41 (80)	48 (92)	.37
Disagree	5 (10)	3 (6)	
**Heart attack associated with smoking**			
Agree	32 (63)	42 (81)	.02
Disagree	15 (29)	6 (12)	
**Stroke associated with smoking**			
Agree	32 (63)	41 (79)	.02
Disagree	13 (26)	4 (8)	
**Sudden infant death syndrome associated with secondhand smoke**			
Agree	16 (31)	35 (67)	<.001
Disagree	23 (45)	9 (17)	
**Ear infections in children associated with secondhand smoke**			
Agree	10 (20)	23 (44)	<.001
Disagree	30 (59)	11 (21)	
**Respiratory infections in children associated with secondhand smoke**			
Agree	41 (80)	47 (90)	.01
Disagree	7 (14)	0	
**Health care providers should advise patients to quit**			
Agree	33 (65)	46 (89)	.01
Disagree	14 (28)	5 (10)	
**Preparedness to help patients quit smoking^b^**			
Highly prepared	9 (18)	15 (29)	.16
Less prepared	38 (75)	32 (62)	
**Ask patients about smoking status**			
Yes	44 (86)	46 (89)	.74
No	7 (14)	6 (11)	
**Recommend patients quit smoking**			
Yes	30 (59)	47 (90)	<.001
No	20 (39)	5 (10)	

a Percentages may not add up to 100 because of missing data.

b Highly prepared: totally or very prepared. Less prepared: somewhat, little, or not at all prepared.
