# Erratum, Vol. 6: Issue 1

**Published:** 2009-03-15

Because of an error in the article "Smoking, Barriers to Quitting, and Smoking-Related Knowledge, Attitudes, and Patient Practices Among Male Physicians in China," which appeared in the January 2009 issue, author Michael C. Fiore was incorrectly identified as having a PhD degree. This designation should have been indicated as an MPH. The correction to the byline was made on January 23, 2009, and appears online at www.cdc.gov/pcd/issues/2009/jan/07_0267.htm. We regret any confusion or inconvenience this error may have caused.

